# The impact of the October 7th war on Israeli medical students’ mental health and well-being

**DOI:** 10.3389/fpubh.2026.1813271

**Published:** 2026-06-24

**Authors:** Jumanah Essa-Hadad, Mary C. J. Rudolf, Lilach Malatskey

**Affiliations:** Azrieli Faculty of Medicine, Bar Ilan University, Safed, Israel

**Keywords:** coping, medical students, mental health, psychological distress, war, well-being

## Abstract

**Background:**

There is a body of literature regarding the high levels of psychological distress routinely experienced by medical students. There is less known about their mental health during times of war and political conflict.

**Objective:**

This study examined the impact of the 2023 October 7th war on Israeli medical students’ mental health, well-being, lifestyle behaviors, academic function and coping strategies.

**Methods:**

A cross-sectional survey with retrospective pre-war self-report of health and academic capability was administered to Bar-Ilan University (BIU) medical students between June and September 2024. Data were collected via an online questionnaire assessing mental health (Depression, Anxiety, and Stress, DASS-21), Kimhi Well-being Scale, Connor-Davidson Resilience Scale, lifestyle behaviors, and coping strategies. Descriptive statistics were conducted; Wilcoxon signed-ranks test was used for pre-post comparisons. Spearman’s rank correlation was used to assess associations between war-related exposure variables and health outcomes.

**Results:**

118 medical students completed the survey. Comparison of health status showed that 96 (81.4%) recalled excellent/very good health pre-war compared to only 45 (38.2%) currently. Mental health distress was substantial: 38 (33.9%) met criteria (above the normal range) for depression, 36 (32.1%) for anxiety and 24 (21.4%) for stress. Sleep quality had declined in 88 (75%) of students, physical activity in 58 (50%) and healthy eating in 71 (61%). Well-being scores fell below adequacy threshold for 69 (59%) of students. In sub-group analysis, male students showed significantly higher resilience scores (*p* = 0.037) than females (Male: M = 39.38, SD = 6.90; Female: M = 36.09, SD = 7.65). Reserve service was significantly associated with poorer mental health status (Spearman’s rank correlation coefficient, *r* = −0.2248, *p* = 0.01). 94 (80%) reported a reduction in ability to study and 72 (61%) poorer academic progress. Primary coping strategies included watching television 67 (58%), spending time with family 65 (57%), and physical activity 54 (47%).

**Conclusion:**

The October 7 war was associated with substantial deterioration in self-reported mental well-being among Israeli medical students. The findings support the need for targeted mental health and resilience-support strategies within medical schools, ideally introduced early. Future research is needed to examine long-term effects and evaluate any interventions.

## 1 Introduction

Medical students are known to encounter high levels of psychological distress during their studies. A 2023 umbrella review and meta-analysis of 32 meta-analyses found that one-third of medical student’s worldwide experience psychological and behavioral symptoms, with highest prevalence for sleep problems, stress, burnout, anxiety and depression (1). Findings have been attributed to the demanding and intense study environment, the hierarchical structure of medical training facilities, and the vulnerability of young adulthood (2).

Less is known about the added impact that war has on medical students’ mental health, although the impact of political conflict and war on general populations is well-documented (3). A cross-sectional study in Syria from 2017 reported alarming rates of psychological distress among medical students in a conflict area, with a prevalence of depression, anxiety, and stress at levels of 60%, 35%, and 53%, respectively (4). Another Syrian study found that 68% of medical students reported stress, with social support from family and friends serving as a significant protective factor (5). Similarly, research from Sudan found that 68% of medical students experienced depression, 58% met criteria for anxiety, and 53% were diagnosed with stress disorder (6). Ukrainian medical students during war demonstrated comparable findings, with 63% reporting stress, 60% anxiety, 59% depression, and 44% exhibiting significant symptoms of post-traumatic stress disorder (7). Academic performance was found to be significantly lower compared to the pre-war period (7). Across all conflict-affected countries, psychological symptoms were more common in women than men (4, 6, 7).

To frame these findings, this study was grounded in the Transactional Model of Stress and Coping (8), which conceptualizes stress as a dynamic process shaped by an individual’s appraisal of a stressor and the coping resources available to manage it. From this perspective, war may influence students’ mental health not only through exposure to external stressors, but also through how they interpret those stressors and mobilize coping strategies and resilience resources. This framework is especially relevant as coping profiles, and social resources may shape well-being during prolonged crises.

An editorial published in 2023 in the International Journal of Medical Students emphasized how political conflicts deeply impact medical students, who are vital to the future of healthcare, technology, and research, underscoring the need to support medical education systems affected by sociopolitical conflicts (9).

The October 7th war generated a profound impact on university students’ mental well-being, highlighting the need for tailored interventions and support services within higher education institutions (10, 11). Our own study during the COVID-19 pandemic demonstrated the vulnerability of medical students to external crises, with significant impacts on mental health and ability to study (12).

During 2024, the October 7th war expanded to areas of Israel other than Gaza, generating an active war zone and evacuation of certain civilian areas in our medical faculty in the north of Israel. This escalation imposed substantially increased stress on the already vulnerable medical student population at Bar-Ilan’s Faculty of Medicine in Northern Israel. While the entire country was impacted, the northern region faced continuous escalation. The Faculty of Medicine is located in Tzfat, 12 km from the Lebanese border, within the primary range of ongoing attacks. According to the Institute for National Security Studies (13), this region sustained thousands of cross-border strikes, resulting in the displacement of over 60,000 residents and an intensity of conflict significantly exceeding the national average. Consequently, the faculty and its teaching hospitals operated under a prolonged state of emergency. This setting is particularly informative because many students have exposure to security threats and military service, providing a unique context for examining responses to acute wartime stress.

Our study aimed to examine the war’s impact on our students’ self-reported mental health, lifestyle, coping strategies and any effect on their ability to study. We hypothesized that students would report deterioration in health behaviors (e.g., sleep, physical activity, and diet) and increased symptoms of depression, anxiety, and stress during the war compared with retrospectively recalled pre-war status. We further hypothesized that the war would have an adverse impact on students’ learning abilities. We were aware of our responsibility as a medical faculty to understand the effect that the war was having in order to consider how best to support our students.

## 2 Methods

### 2.1 Study design

A cross-sectional survey was employed to examine the impact of the October 7th war on medical students’ levels of stress, anxiety, depression, well-being, lifestyle behaviors, academic function and the coping strategies they employed (Table 1). All data were collected at a single time point during the war, and students were asked to retrospectively report their pre-war health status and behaviors. The study was conducted between June 2024 and September 2024. Approval was attained from the BIU Faculty of Medicine Ethics in Research Committee, approval number 05-2024.

**Table 1:** Self-reported health status (*n* = 118) and mental health status (*n* = 112) before and during war.

	Rating	Before war	During war	*p*	*r_s_*
Self-reported perceived health status (*n* = 118)	Excellent	54 (46.2%)	16 (13.7%)	<0.001	0.73
	Very good	41 (35.0%)	29 (24.8%)		
	Good	21 (18.0%)	47 (40.2%)		
	Not so good	0	23 (19.7%)		
	Bad	2 (1.7%)	3 (2.6%)		
Self-reported perceived mental health status (*n* = 112)	Excellent	34 (30.4%)	4 (3.6%)	<0.001	0.87
	Very good	50 (44.6%)	21 (18.8%)		
	Good	25(22.3%)	43 (38.4%)		
	Not so good	1 (0.9%)	36 (32.1%)		
	Bad	2 (1.79%)	8 (7.1%)		

*Before war and during war ratings were compared using the Wilcoxon signed-rank test; r_s_ denotes the Spearman correlation coefficient reported as a measure of effect size (r_s_).

### 2.2 Setting and participants

The research was conducted at the Azrieli Faculty of Medicine, Bar-Ilan University, established in 2011 and located in Tzfat, a city in Israel’s disadvantaged Northern periphery. The medical school operates as a remote campus, with the main university campus over 165 kilometers away in Ramat Gan. This creates various challenges, including limited social opportunities and reduced access to facilities compared to main campus programs.

Three MD programs are offered: a 4-year program for students with a prior degree, a 3-year program for students who studied the first 3 years of medical school abroad, and a 6-year program for students directly following high school/army service. Clinical education takes place at six affiliated hospitals across the region.

The medical student body comprises medical students who are mostly Jewish and religiously secular, with a predominance of women. With one program of our medical school being post-graduate, as well as most students completing military service before starting university (Table 2), our students tend to be older than medical school populations elsewhere, with about two-thirds of them married or in long-term relationships and 20% with children.

**Table 2:** Changes in health behaviors and academic function during war (compared to pre-war self-report), frequencies and Wilcoxon tests.

Behavior	Sig. more *n* (%)	Somewhat more *n* (%)	No change *n* (%)	Somewhat less *n* (%)	Sig. less *n* (%)	Media*n*	*p*	*r*
Exercise (*n* = 117)	10 (8.5%)	14 (12.0%)	35 (29.9%)	28 (23.9%)	30 (25.6%)	3.0	<0.001	0.31
Healthy eating (*n* = 117)	4 (3.4%)	11 (9.4%)	31 (26.5%)	39 (33.3%)	32 (27.4%)	4.0	<0.001	0.50
Smoking (n = 100)	11 (11.0%)	14 (14.0%)	69 (69.0%)	2 (2.0%)	4 (4.0%)	3.0	0.144	0.15
Alcohol use (*n* = 110)	9 (8.2%)	13 (11.8%)	77 (70.0%)	5 (4.5%)	6 (5.5%)	3.0	0.747	0.03
Sleep quality (*n* = 117)	1 (0.9%)	5 (4.3%)	23 (19.7%)	42 (35.9%)	46 (39.3%)	4.0	<0.001	0.56
Academic studies								
Ability to study (*n* = 117)	0 (0.0%)	5 (4.3%)	18 (15.4%)	42 (35.9%)	52 (44.4%)	4.0	<0.001	0.61
Academic success (*n* = 117)	1 (0.9%)	6 (5.1%)	38 (32.5%)	46 (39.3%)	26 (22.2%)	4.0	<0.001	0.53

The research targeted 380 medical students studying in Bar Ilan Faculty of Medicine in all three MD programs in both preclinical and clinical years enrolled at the university at the time of the study. Of the 380 medical students who were sent the questionnaire, 118 (31%) responded. Post-hoc sensitivity analysis confirmed that our sample size of 118 provided 80% power to detect medium-sized effects (Cohen’s *d* = 0.5) in paired comparisons at alpha = 0.05.

### 2.3 Survey instruments

The survey consisted of multiple components.

#### 2.3.1 Personal questionnaire (27-items)

**Figure 1:**
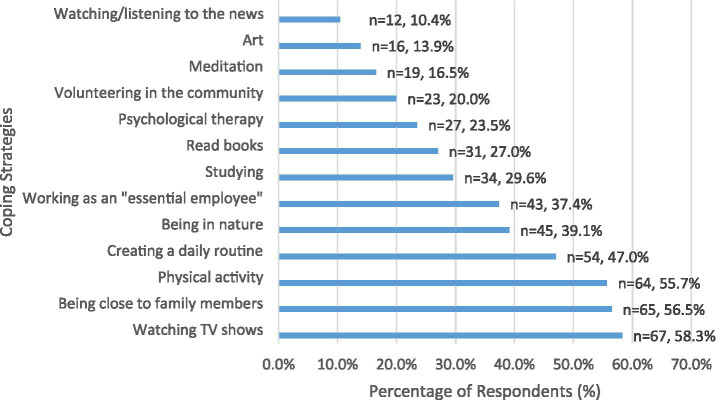
Strategies medical students used to cope with stress during the war.

A questionnaire that we had originally developed during the COVID epidemic (12) was revised to evaluate medical students’ lifestyle and concerns during the war (Figure 1). Because this study was cross-sectional, pre-war health could not be measured prospectively but relied on recalled baseline status. It included change in health status before and during the war; change in lifestyle behaviors, namely physical activity, adherence to a healthy diet, sleep, smoking and alcohol consumption; learning efficacy and academic progress and sociodemographic parameters. The scale employed a five-level subjective scale (excellent, very good, good, not so good, usually not good). Students were also asked about coping strategies they employed to help cope with stress and any recommendations they had regarding how the Faculty might support them during times of crisis.

#### 2.3.2 Kimhi well-being scale

This 9-item questionnaire, validated in Hebrew, was used to assess overall well-being, with scores ranging from 1 to 6. A score falling below the adequacy threshold (<35) indicates inadequate well-being (14). Internal consistency for the Kimhi Well-being Scale was high (Cronbach’s *α* = 0.89, 95% CI [0.86, 0.92]), indicating strong reliability within our sample.

#### 2.3.3 Connor-Davidson resilience scale (CD-RISC)

The validated short 10-item version of the CD-RISC was used to assess students’ resilience, with a score <32 indicating inadequate resilience (15). For the CD-RISC-10, the Cronbach’s alpha was 0.91 (95% CI [0.86, 0.94]), indicating excellent internal consistency.

#### 2.3.4 Depression, anxiety, and stress scale (DASS-21)

Psychological distress was assessed using the validated Hebrew version of the DASS questionnaire consisting of 21 items, 7 items in each section (16). It evaluates three domains, depression, anxiety, and stress, with established cutoff scores for severity classification (normal, mild, moderate, severe, extremely severe). The Cronbach’s alpha for DASS-21 was 0.95, indicating excellent internal consistency (*α* = 0.95, 95% CI [0.92, 0.96]).

### 2.4 Data collection

Students were invited to participate in the study by e-mail sent from the Faculty’s administrator and the student body with a link to the anonymous and secure online survey via the Google Survey platform. Before entering the survey, participants were asked to provide online consent. Upon completion of the survey, students received a message indicating that they could send a separate email with their name and contact details to participate in a lottery. Ten gift vouchers for coffee were offered as incentives.

### 2.5 Statistical analysis

Descriptive statistics were performed using the Statistical Package for the Social Sciences software version 26 (SPSS Inc., Chicago, IL, USA). Change in self-reported health was assessed based on students’ retrospective recall of pre-war health status compared to their current self-report during the war. Wilcoxon signed-rank tests were conducted to determine statistically significant changes, taking a *p*-value of <0.05 as significant. To examine differences in demographic characteristics (gender and relationship status) and association with exposure variables (reserve service, trauma exposure), we performed subgroup analysis on primary outcomes including DASS-21 scores, Kimhi Well-being scores, and CD-RISC resilience scores. For the DASS-21, higher scores equal greater distress while for Kimhi well-being/CD-RISC, higher scores indicate better well-being/resilience. We used independent sample t-tests for demographic comparisons and Spearman’s correlation coefficient to assess association between trauma exposure and reserve status and perceived health and mental health status. For these analyses, reserve service was coded on a 4-point ordinal scale (1 = not in reserves; 2 = up to 1 month; 3 = up to 3 months; 4 = more than 3 months), and trauma exposure on a 5-point scale (1 = no exposure; 2 = distant friends; 3 = close friends/family; 4 = personally injured; 5 = death). Perceived health and mental health status was coded on a 5-point Likert scale where 1 indicated ‘excellent’ and 5 indicated “bad”.

## 3 Results

A total of 118 students (31%) completed the survey, 78 (66%) were female; 67 (57%) were married or in a relationship. The majority, 109 (93%), reported they were Jewish, with remaining students reporting they were Muslim, Druze, or Atheist. There were 23 (19.5%) students who reported having at least one child.

Twenty-one (17.8%) students had been evacuated during the war - 8 for up to 1 month, 9 for 3 months, and 4 for more than 3 months. Only 34 students (29.1%) reported no direct trauma exposure; 50 (42.7%) knew more distant friends or acquaintances who were directly affected (either displaced or in the army reserves), 13 (11.1%) had close friends affected, and 10 (8.5%) experienced the death of someone they knew.

Forty-one (34.7%) reported service in the military reserves; 4 (3.5%) served for up to 1 month, 10 (8.7%) for up to 3 months, and 24 (20.9%) were currently active in the reserves. Reserve service in spouses was less frequent; Five (4.3%) served up to 1 month, 9 (7.8%) up to 3 months, and 15 (12.9%) remained in the reserves.

Eighty-two students (69.2%) reported community volunteer engagement: 48 (41.0%) volunteered for up to 1 month, 20 (17.1%) for up to 3 months, and 13 (11.1%) remained active, whereas 36 (30.8%) did not volunteer.

War-related worries and concerns were prevalent across multiple dimensions: 39 students (33.1%) were significantly worried about their economic situation, 49 (41.5%) worried to an extent, 19 (16.1%) slightly worried, and only 11 (9.3%) were not worried. Thirty-three students (28%) were significantly worried about personal injury or death, and 54.8% (*n* = 63) about a family member or friend being harmed.

### 3.1 Perceived health and mental health status

Perceived health status ratings declined markedly according to students’ recall of their health pre-war and their current report of health as shown in Table 1. A Wilcoxon signed-rank test revealed that the decline was statistically significant (*p* < 0.001). Three students indicated improved health status, and 34 students had no change.

Perceived mental health status also declined significantly (Wilcoxon signed rank test, *p* < 0.001, *r* = 0.87) from before the war to during. The proportion of students rating their mental health as excellent or very good dropped from almost 75% (*n* = 84) to 23% (*n* = 25).

Reserve service was significantly associated with poorer mental health status (Spearman’s rank correlation coefficient, *r* = −0.2248, *p* = 0.01). Since higher DASS-21 scores reflect greater distress, the negative correlation indicates that students who experienced reserve service reported higher levels of depression, anxiety, and stress.

Trauma exposure showed no association with mental health status (Spearman’s rank correlation coefficient, *r* = −0.035, *p* = 0.709).

### 3.2 Self-reported health behaviors

Self-reported health behaviors shifted in a downward direction during the war. Fifty-eight (49.2%) reported decrease in exercise, 71 (60.1%) in healthy eating, 88 (74.6%) in sleep quality. Wilcoxon signed-rank tests confirmed decline was statistically significant for exercise (*p* = 0.001, *r* = 0.31), healthy eating (*p* < 0.001, *r* = 0.50), and sleep quality (*p* < 0.001, *r* = 0.56). This was accompanied by 25 students (21.2%) who smoked more and 22 (18.6%) who consumed more alcohol; however, these changes were not statistically significant.

Students reported that the war had a negative impact on their academic function. Reduced ability to study was most prevalent (*n* = 94, 79.7%), with statistically significant decline confirmed (*p* < 0.001, *r* = 0.61), alongside poorer progress (*n* = 72, 61%, *p* < 0.001, *r* = 0.53). Worry of academic failure affected 76.6% of students.

### 3.3 Well-being and resilience

Well-being scores fell below the adequacy threshold (< 35) for 64 (55%) students; the mean score for well-being was only 31.7 (SD 8.5). No statistically significant differences were seen between males and females for the Kimchi Well-being score.

Among the 112 students who completed the CD-RISC-10, 4 (3.6%) demonstrated low resilience, 49 (43.8%) moderate resilience, and 59 (52.7%) showed high resilience. In sub-group analysis, male students showed significantly higher resilience scores (*p* = 0.037) than females (Male: M = 39.38, SD = 6.90; Female: M = 36.09, SD = 7.65).

### 3.4 Depression, anxiety, and stress (DASS-21)

Table 3 shows the results of the 112 students who completed DASS-21. Thirty-eight students (33.9%) met the criteria for depression of whom 20 were mild, 16 moderate and two severe. Thirty-six students (32.1%) met the criteria for anxiety, of whom 8 were mild, 21 moderate, 6 severe, and one extremely severe. Twenty-four students (21.4%) met the criteria for stress, including 12 mild and 12 moderate cases. DASS-21 total scores showed no statistically significant differences (male: mean (SD) 42.1 (14.5); female: 46.3 (14.63); *p* = 0.168). No statistically significant difference in DASS-21 was found for students in a relationship (mean (SD): 43.7 (14.2) vs. 46.3 (15.2), *p* = 0.314).

**Table 3:** Depression, anxiety, and stress scores among medical students during the war (*n* = 112).

DASS-21 category	Depression	Anxiety	Stress
Normal	75 (66.4%)	77 (68.1%)	89 (78.8%)
Mild	20 (17.7%)	8 (7.1%)	12 (10.6%)
Moderate	16 (14.2%)	21 (18.6%)	12 (10.6%)
Severe	2 (1.8%)	6 (5.3%)	0 (0%)
Extremely severe	0 (0%)	1 (0.9%)	0 (0%)

### 3.5 Coping strategies

Students employed diverse coping strategies to manage war-related stress: 67 students (58.3%) watched TV shows, 65 (56.5%) spent time with family, and 54 (47.0%) both engaged in physical activity and established a daily routine, with most students reporting three or more different strategies.

## 4 Discussion

Our study examined the impact of the 2023 October 7th war on Israeli medical students’ mental health, well-being, lifestyle behaviors, academic function and coping strategies. As anticipated, the war had a substantial adverse effect on students’ mental health, well-being and academic functioning. These findings affirmed that even in a population with historical exposure to political conflicts, acute wartime conditions generate marked psychiatric impact.

The study found higher resilience scores among male students, but no significant gender difference in DASS-21 or well-being. When we examined war-related exposure variables, trauma exposure was not associated with current mental health status, whereas reserve service was significantly associated with poorer mental health, suggesting that different forms of war exposure have different psychological effects, with reserve duties potentially representing a more sustained source of stress.

Compared with other conflict-affected countries, our participants reported lower rates of depression, anxiety, and stress. In 2025, Ukrainian medical students documented 62.5% reporting stress, 59.6% anxiety, and 58.8% depression (7). Similarly, Syrian medical students (in 2017) and Sudanese medical students (in 2025) showed prevalence rates of 61% and 68% for depression, 35.1 and 58% for anxiety, and 52.6% and 53% for stress (4, 6).

The relatively lower rates in our study may reflect factors such as Israel’s prior experience with security threats, the age and life circumstances of our students, and the presence of family and social support. Our students are older than typical medical students, with many married or in long-term relationships and some having children; most have had military experience in the past that may have tempered their response to military events. These features likely have mixed effects on self-perceived stress, anxiety, and depression: family responsibilities and concern for spouses or children may intensify emotional strain, while partner and family support may buffer distress for some students. However, these explanations remain speculative and were not directly tested in this study.

A striking finding was the marked decline in perceived health and mental health status: the proportion of students rating health as excellent or very good decreased by more than half representing a severe reduction in subjective well-being. This decline was paralleled by significant deterioration in health behaviors, particularly sleep where over 70% reported decreased sleep quality and reduced academic engagement, half exercised less, and two thirds ate less healthily. Lifestyle behaviors were not universally affected for the worse. Some students reported improvement in lifestyle, particularly increased physical activity, which they may have used as a way to cope with the stress of the war. Students employed various coping strategies, the most common included watching movies/TV shows, being close to family members, and physical exercise. Interestingly, medical students reported coping strategies similar to those reported during the COVID-19 pandemic (12).

### 4.1 War versus the COVID-19 pandemic

To contextualize our findings, we compared our findings with mental health data collected during the COVID-19 pandemic (12). The findings were not dissimilar with 40% of students meeting depression criteria compared with 34% during the war, 25% meeting anxiety criteria compared with 32% during the war, and 27% meeting stress criteria compared to 21% during the war. However, critical differences emerged in severity. During the pandemic, 12% met criteria for severe depression, 10% for severe anxiety and 11% for severe stress compared with 2, 6% and 0 during the war. This pattern suggests that Israeli students reported a different profile of mental health symptoms during wartime compared to the unprecedented global disruption of the pandemic. While the prevalence of distress was broadly similar, the lower proportion of students meeting criteria for severe distress during the war suggests that the two crises may have influenced student well-being through different mechanisms. It should be noted, however, that the two studies differ in sample characteristics, timing, and context and comparison between the two circumstances should be undertaken with caution.

The pandemic generated uncertainty about some fundamental aspects of daily life, education, social interaction, and healthcare, affecting all aspects of existence. Perhaps war, while undoubtedly stressful, feels more “manageable” within the context of Israeli collective experience. The pandemic’s enforced isolation certainly disrupted the family and social connection mechanisms that appear to serve as protective factors in wartime (as evidenced by so many students reporting family time as a coping strategy).

### 4.2 Implications for medical schools and recommendations

Addressing psychological distress requires multi-faceted approaches. Evidence suggests that for students in higher education, mental health care, social support, decreased academic duties, and providing more free time are essential protective measures (17). In addition, understanding and enhancing coping strategies as a tool for students to deal with stress is important. Research on American military medical students identified key coping mechanisms such as social connection, exercise, relaxation, and work-life balance (18).

Our findings indicate that large-scale crises of this kind not only harm students’ mental health but also impair their capacity to learn and engage with the curriculum, thereby undermining the core mission of medical schools to train future physicians. This dual impact provides a strong rationale for institutional investment in structured support and for the development of explicit crisis-response frameworks within medical education. Rather than assuming that coping resources will emerge spontaneously, schools should integrate evidence-informed resilience and mental health–promoting strategies from the outset of training, with the capacity to intensify academic and psychosocial support during periods of acute stress (19). The literature suggests several evidence-based recommendations to support medical students, including sleep hygiene programs to address sleep decline, integrating physical activity components into medical training, strengthening peer support and mentorship networks to facilitate social connections, brief cognitive-behavioral and mindfulness-based interventions to promote resilience and reduce distress (20–24).

Based on our findings of substantial psychological distress and the broader literature on medical student well-being, we identify several modifiable risk factors that medical schools can target to improve outcomes. These include addressing factors such as academic burden, which was supported by our data, and other factors like poor social relationships, living alone, and financial hardships, which are well-established areas for institutional intervention in the literature (17, 21). Targeted interventions and support services might reduce the risk of long-term mental morbidity. We are currently planning to develop a screening index tool for students’ mental and emotional well-being, which will be administered to all medical students once a year and at times of crisis such as war, to identify those in distress and proactively invite them to receive appropriate support and care.

Israeli culture emphasizes family connection and social solidarity, which we identified as coping strategies. These values are likely to be evident elsewhere and medical schools might leverage these cultural strengths by also facilitating family involvement, strengthening peer cohort identity, and emphasizing collective support during training.

### 4.3 Strengths and limitations of our study

Our study has several strengths. The data was collected during the ongoing conflict, offering a real-time snapshot of students’ health and well-being. We used validated mental health questionnaires, giving a more reliable understanding of the influence on mental health, and also enquired about a broad set of variables, including lifestyle behaviors, perceived health, and effect on learning. This provided a multi-dimensional approach rather than focusing on a single outcome.

However, some limitations of the current study need to be acknowledged. The response rate was lower than we had hoped. Questionnaire fatigue is a common phenomenon among medical students, and this was no doubt compounded by stressful circumstances. It may therefore be considered as something of an achievement. But it does raise the potential of response bias, although the direction of any bias is uncertain as distressed students may be more or less inclined to complete a survey of this nature.

Another limitation is the retrospective nature of our pre-war health, lifestyle, and academic assessments, which may have introduced recall bias. Because this study was cross-sectional, pre-war health could not be measured prospectively but relied on recalled baseline status. Additionally, our findings rely exclusively on self-reported measures rather than objective clinical assessments. This reliance may introduce various forms of response bias, including social desirability, recall bias, and systematic under- or over-reporting, which can limit the objective characterization of mental health diagnoses.

The lack of data on mental health status and fear of failure prior to the war or pandemic is another study limitation. It would have been interesting to obtain actual measures of academic progress, but due to anonymity of the survey, this was not possible.

Although individual-level data on non-responders were not available because the survey was anonymous, the sample also needs to be considered in the context of the wider medical student body, which is predominantly female, older, and religiously secular Jewish. These features are of relevance in considering the generalizability of the findings. Finally, the study was conducted in a single medical school in a specific regional and sociopolitical context, which limits generalizability of the findings.

## 5 Conclusion

The October 7th war was associated with an adverse psychological impact on Israeli medical students, with approximately one-third meeting the clinical criteria for depression or anxiety, and one-fifth for stress. Students reported significant deterioration in health behaviors, ability to study and well-being. These findings underscore the critical need for interventions implemented at both individual and institutional level.

Medical schools should consider proactively addressing mental health needs before crises occur. Based on our findings, we suggest that comprehensive resilience-building programs be implemented from medical school entry, incorporating cognitive-behavioral training, mindfulness instruction, physical activity promotion, structured social connection, and targeted reduction of modifiable risk factors including academic overload, financial hardship, and social isolation.

Future research should employ prospective longitudinal designs, involving multiple locations for enhanced generalizability, and integrate objective outcome measures including academic performance data. Qualitative research exploring student-identified needs and institutional resources would further inform implementation of evidence-based interventions.

## Data Availability

The raw data supporting the conclusions of this article will be made available by the authors, without undue reservation.

## References

[1] Jahrami H AlKaabi J Trabelsi K Pandi-Perumal SR Saif Z Seeman MV . The worldwide prevalence of self-reported psychological and behavioral symptoms in medical students: an umbrella review and meta-analysis of meta-analyses. J Psychosom Res. (2023) 173:111479. doi: 10.1016/j.jpsychores.2023.111479, 37651841

[2] Mengesha AK Misker MF Tadesse SA. Identifying factors contributing to depression and anxiety among medical students: a multicenter cross-sectional study. Sci Rep. (2025) 15:33792. doi: 10.1038/s41598-025-99177-4, 41028282 PMC12484637

[3] Charlson F van Ommeren M Flaxman A Cornett J Whiteford H Saxena S. New WHO prevalence estimates of mental disorders in conflict settings: a systematic review and meta-analysis. Lancet. (2019) 394:240–8. doi: 10.1016/S0140-6736(19)30934-1, 31200992 PMC6657025

[4] Al Saadi T Zaher Addeen S Turk T Abbas F Alkhatib M. Psychological distress among medical students in conflicts: a cross-sectional study from Syria. BMC Med Educ. (2017) 17:173. doi: 10.1186/s12909-017-1012-2, 28931387 PMC5607487

[5] Al Houri HN Jomaa S Arrouk DMN Nassif T Al Ata Allah MJ Al Houri AN . The prevalence of stress among medical students in Syria and its association with social support: a cross-sectional study. BMC Psychiatry. (2023) 23:97. doi: 10.1186/s12888-023-04593-3, 36750821 PMC9906887

[6] Alfadul ESA Alrawa SSK Hemmeda L Adam AYT Mohamed Salih Mohamed Nour S Ebrahim Hamed Saeed N . Effect of military conflict on mental health: a cross-sectional study among the medical students at Khartoum governmental universities, Sudan, 2023. BMJ Open. (2025) 15:e086495. doi: 10.1136/bmjopen-2024-086495, 40132815 PMC11956355

[7] Korda M Shulhai A Shevchuk O Shulhai O Shulhai AM. The psychological well-being and academic performance of Ukrainian medical students under the burden of war: a cross-sectional study. Front Public Health. (2025) 12:1457026. doi: 10.3389/fpubh.2024.1457026 39835319 PMC11743275

[8] Lazarus RS Folkman S. Stress, Appraisal, and Coping. New York, NY: Springer Publishing Company (1984).

[9] Takoutsing BD Găman M-A Puyana JC Bonilla-Escobar FJ. The silent casualties: war’s impact on medical students and medical education. Int J Med Stud. (2023) 11:254–8. doi: 10.5195/ijms.2023.2476

[10] Dobiesz VA Schwid M Dias RD Aiwonodagbon B Tayeb B Fricke A . Maintaining health professional education during war: a scoping review. Med Educ. (2022) 56:793–804. doi: 10.1111/medu.14808, 35388529 PMC9540571

[11] Dopelt K Houminer-Klepar N. War-related stress among Israeli college students following 7 October 2023 terror attack in Israel. Eur J Investig Health Psychol Educ. (2024) 14:2175–86. doi: 10.3390/ejihpe14080145, 39194939 PMC11353874

[12] Peer M Rudolf MCJ Essa-Hadad J Malatskey L. The effects of the COVID-19 pandemic on the mental health and learning abilities of medical students: a cross-sectional study. JIMS. (2021) 1:8–15.

[13] Institute for National Security Studies (INSS). Swords of Iron: Key Data and Analysis of the Northern Front. Tel Aviv: Institute for National Security Studies (INSS) (2024).

[14] Kimhi S Eshel Y Marciano H Adini B. Distress and resilience in the days of COVID-19: comparing two ethnicities. IJERPH. (2020) 17:3956. doi: 10.3390/ijerph17113956, 32503205 PMC7312505

[15] Connor KM Davidson JR. Development of a new resilience scale: the Connor-Davidson resilience scale (CD-RISC). Depress Anxiety. (2003) 18:76–82. doi: 10.1002/da.10113, 12964174

[16] Lovibond SH Lovibond PF. Manual for the Depression, Anxiety, Stress, Scale. Sydney: Psychology Foundation (1995).

[17] Hyseni Duraku Z Davis H Hamiti E. Mental health, study skills, social support, and barriers to seeking psychological help among university students: a call for mental health support in higher education. Front Public Health. (2023) 11:1220614. doi: 10.3389/fpubh.2023.1220614, 37920583 PMC10619655

[18] Ma TL Bell K Dong T Durning SJ Soh M. Military medical students' coping with stress to maintain well-being. Mil Med. (2023) 188:26–34. doi: 10.1093/milmed/usac292, 37201497

[19] Sperling EL Manion B Gresner E LaBarre A Krych P Mallender ZC. The effect of resilience training on resilience and stress in medical students: a systematic review and meta-analysis. BMC Med Educ. (2025) 26:7. doi: 10.1186/s12909-025-08171-x, 41316127 PMC12771728

[20] Sahranavard S Esmaeili A Salehiniya H Behdani S. The effectiveness of group training of cognitive behavioral therapy-based stress management on anxiety, hardiness and self-efficacy in female medical students. J Educ Health Promot. (2019) 8:49. doi: 10.4103/jehp.jehp_327_18, 30993142 PMC6432834

[21] Wasson LT Cusmano A Meli L Louh I Falzon L Hampsey M . Association between learning environment interventions and medical student well-being: a systematic review. JAMA. (2016) 316:2237–52. doi: 10.1001/jama.2016.17573, 27923091 PMC5240821

[22] Ziegelstein RC. Creating structured opportunities for social engagement to promote well-being and avoid burnout in medical students and residents. Acad Med. (2018) 93:537–9. doi: 10.1097/ACM.0000000000002117, 29280756

[23] García-Pérez L Atencia-Rodriguez ME Cepero-González M Padial-Ruz R. Effectiveness of physical activity, mindfulness and mind-body therapies in improving mental health of university students: a systematic review of RCTS. J Am Coll Heal. (2025) 74:1085–100. doi: 10.1080/07448481.2025.2492174, 40262195

[24] A Mariappan V Mukhtar F. Psychological interventions for sleep problems among medical and paramedical students: a systematic review. J Clin Psychol. (2025) 81:399–409. doi: 10.1002/jclp.23781 40056459

